# The combination of dantrolene and nimodipine effectively reduces 5-HT-induced vasospasms in diabetic rats

**DOI:** 10.1038/s41598-021-89338-6

**Published:** 2021-05-10

**Authors:** Marie Román, Laura García, Myrna Morales, María J. Crespo

**Affiliations:** 1 Department of Physiology, University of Puerto Rico-School of Medicine, GPO Box 365067, San Juan, PR 00936-5067 USA; 2 Department of Anesthesiology, University of Puerto Rico-School of Medicine, GPO Box 365067, San Juan, PR 00936-5067 USA

**Keywords:** Translational research, Pharmacology

## Abstract

Diabetics have a higher risk of developing cerebral vasospasms (CVSP) after subarachnoid hemorrhagic stroke than non-diabetics. Serotonin (5-HT) is one of the key vasoconstrictors released in the hemorrhagic blood and an important contributor to the etiology of CVSP. The combination of the ryanodine receptor blocker dantrolene and the Ca2+ channel blocker nimodipine significantly reduces phenylephrine (PHE)-induced vascular contraction in both diabetic and nondiabetic rats, but the effectiveness of this drug combination in reducing 5-HT-induced contraction is unknown. Dose–response curves for the 5-HT-induced contraction (from 0.1 nM to 100 µM) were performed on aortic rings from diabetic and non-diabetic rats after a 30-min incubation period with dantrolene, nimodipine, and both drugs in combination. In diabetic rats, 10 μM of dantrolene alone failed to reduce 5-HT-induced maximal contraction (E_max_), but 50 μM reduced this parameter by 34% (n = 7, p < 0.05). In non-diabetic rats, by contrast, dantrolene did not modify the vascular response to 5-HT. 50 nM of nimodipine alone, however, reduced this parameter by 57% in diabetic rats (n = 10, p < 0.05), and by 34% in non-diabetic rats (n = 10, p < 0.05). In addition, concomitant administration of dantrolene and nimodipine reduced vascular reactivity to a similar extent in both diabetic (~ 60% reduction, n = 10, p < 0.05) and non-diabetic rats (~ 70% reduction, n = 10, p < 0.05). Moreover, the combination of nimodipine with the higher concentration of dantrolene significantly increased the EC_50_ values for the 5-HT-induced contraction curves in both diabetics (from 10.31 ± 1.17 µM to 19.26 ± 2.82; n = 10, p < 0.05) and non-diabetic rats (5.93 ± 0.54 µM to 15.80 ± 3.24; n = 10, p < 0.05). These results suggest that simultaneous administration of dantrolene and nimodipine has a synergistic effect in reducing 5-HT-induced vascular contraction under both diabetic and non-diabetic conditions. If our findings with rats are applicable to humans, concomitant administration of these drugs may represent a promising alternative for the management of CVSP in both diabetics and non-diabetics.

## Introduction

Subarachnoid hemorrhage (SAH) has a higher morbidity and mortality rate than any other type of stroke^1^. Approximately 70% of SAH patients develop severe cerebral vasospasms (CVSPs), although evident neurological deficit appears in only 30% of this population^2^. This neurological deterioration results from the delayed cerebral ischemia that is secondary to potent vasoconstriction, which results from increased intracellular Ca2+ levels in vascular smooth muscle (VSM)^3^. The underlying mechanisms are unknown, but serotonin (5-hydroxytriptamine, 5-HT), endothelin-1, sphingosine, oxyhemoglobin, and norepinephrine, have been implicated in the etiology of CVSPs because all these vasoconstrictors may promote vascular hyper-reactivity by increasing intracellular Ca2+ in VSM^4–6^.

The incidence of subarachnoid hemorrhage (SAH) is higher in diabetics than in non-diabetics because diabetics are more likely to develop CVSPs, even when under glycemic control^7^. Although the pathophysiology may differ between diabetics and non-diabetics, the existing treatment is similar for the two groups. The current pharmacological approach includes the administration of Ca2+ channel blockers (CCB), to prevent Ca2+ entry through L-type voltage dependent Ca2+ channels (VDCC). Nevertheless, despite standard therapies following CVSP, multiple complications remain, including neurological deficits (34%), delayed cerebral ischemia (30%), and death (30%)^8–10^. In non-diabetics, concomitant administration of a CCB and dantrolene, a ryanodine receptor (RyR) blocker that inhibits Ca2+ release from the sarcoplasmic reticulum, improves outcomes due to the ability of this combination to reduce vasoconstriction^3,11–13^. Whether the combination of a CCB with dantrolene will also be effective for diabetics remains unknown. Thus, considering that hyperglycemia alters RyR and VDCC regulation and expression^14,15^, it is essential to determine if adding dantrolene to standard CCB therapies helps to reduce morbidity and mortality in diabetic patients with CVSP.

Previous studies of diabetic and non-diabetic rats in our laboratory indicated that concomitant administration of dantrolene and the CCB nimodipine significantly lessens vascular tone by reducing phenylephrine (PHE)-induced contraction of aortic rings^16,17^. The effects of this drug combination on the 5-HT-induced vascular response in diabetics, however, remain unknown. 5-HT is one of the most important endogenous neurotransmitters in the regulation of vascular tone and blood flow; its effects at the vascular level are dependent on Ca2+ trafficking through VSM^18^. Under pathological conditions, such as hypertension or diabetes, 5-HT produces an abnormally enhanced vascular contraction^19,20^. In SAH, 5-HT is produced in the hemorrhagic blood and is implicated in the genesis of the potent vasospasm occurring in the cerebral vasculature 4 to 14 days after the hemorrhage^10,21,22^. Thus, owing to the need for developing new pharmacological approaches to treat diabetics with CVSP, using a diabetic animal model to characterize vascular responses to drug combinations with the potential for reducing intracellular Ca + 2 in VSM is critical to the design of more effective therapies. In the current study, we investigate the effects of dantrolene, either alone or in combination with nimodipine, on the 5-HT-induced contractions of aortic rings from streptozotocin (STZ)-induced diabetic rats.

## Materials and methods

### Experimental animal model

Forty male Sprague–Dawley rats (Taconic Biosciences Inc, Germantown, NY, USA), approximately four weeks of age, were divided into two groups: diabetic and non-diabetic (control), with each group consisting of 40 animals. To induce diabetes, rats were fasted overnight and then injected intraperitoneally (IP) with streptozotocin (STZ; 65 mg/kg) dissolved in a 0.1 M citrate buffer, having a pH of 4.5. This methodology has been previously described by Quidgley and collaborators^25^. The diabetic rats never received insulin supplementation. Non-diabetic animals were only injected with the citrate buffer solution. Hyperglycemia was verified 24 h after the STZ-injection with a TRUEtrack blood glucose monitoring system (NIPRO Diagnostics, Fort Lauderdale, FL, USA). Blood glucose levels were monitored once a week in all animals. All experiments were performed at four weeks after induction of diabetes. Animals were housed in a temperature-controlled room with unlimited access to water and food (Harlan Rodent Diet, 18% protein). All procedures involving the animals were approved by the Institutional Animal Care and Use Committee (Protocol #2,590,115), and adhered to the Guide and Care for the Use of Laboratory Animals published in 2011 by the National Institutes of Health (USA). In addition, all the experiments and methods carried out in this study were in compliance with the Animal Research: Reporting of In Vivo Research (ARRIVE) guidelines.

### Tissue preparation for isometric tension studies

To evaluate 5-HT-induced contraction, we followed the protocol previously described by our laboratory^25^. Briefly, rats were first anesthetized with a combination of ketamine (50 mg/kg, IP) and xylazine (4 mg/kg, IP). Once anesthesia was achieved, 5 mm segments were obtained from the proximal portion of each aorta. Avoiding damage to the smooth muscle and endothelium, the connective tissue adjacent to the aortic adventitia was carefully removed. The aortic segments were placed in a two-hook, 50 ml chamber (Radnoti Co, Monrovia, CA, USA), containing a Krebs' bicarbonate solution bubbled with a mixture of 95% O2 and 5% CO2 at 37 °C. A resting tension of 2.3 g. was applied to the rings, and then they were connected to a FT03C Grass transducer (Warwick, RI, USA) and equilibrated by one hour. A data acquisition card (National Instruments, Austin, TX, USA; PC-LPM-16/PnP) and the LabView software from National Instruments (Austin, TX, USA) were used to analyze and record changes in isometric tension.

### Measurement of aortic ring 5-HT-induced contraction

The effects of 10 µM and 50 µM dantrolene, 50 nM nimodipine, and these two drugs in combination, on the 5-HT-induced contraction were evaluated following the protocols previously described by our laboratory^16,17^. Cumulative concentration–response curves were generated by adding 5-HT (0.1 nM to 100.0 μM) to the aortic rings before and after incubation with the drugs. The values of Emax (maximum contraction) and EC50 (concentration required to reach 50% of maximum ontraction) were obtained by analyzing the concentration–response curves for each group.

### Drugs

Dantrolene, nimodipine, phenylephrine (PHE), acetylcholine (Ach), sodium nitroprusside (SNP), and NG-nitro-L-arginine (L-NAME) were obtained from Sigma Chemical Co. (Saint Louis, MO, USA). The selected concentrations of nimodipine (50 nM) and dantrolene (10 µM and 50 µM) were based on comparable studies of different vascular beds in rats^5,23,24^.

## Statistical analysis

Data were presented as the mean ± SEM of five to nine animals per group. Statistical differences between groups were analyzed using the 2-tailed unpaired Student’s t-test. When more than two groups were compared, ANOVA was applied, followed by the post-hoc Student–Newman–Keuls test. The D’Agostino and Pearson omnibus normality test revealed that the data were normally distributed. In addition, the non-linear regression curve fit from GraphPad Prism 5 found no outlying data points. Differences were considered significant when p ≤ 0.05.

## Results

Tables 1 and 2 illustrate the blood glucose values and weight of the experimental animals. In non-diabetic rats, blood glucose concentration ranged from 108.4 ± 4.3 to 86.02 ± 2.9 mg/dl throughout the study. In diabetic rats, however, glucose concentrations were higher, ranging from 518 ± 11.8 mg / dl at 24 h after STZ administration to 576.8 ± 11.9 mg / dl at the end of the study period. In addition, body weight remained significantly lower in diabetics (224.8 ± 11.0 g) than in non-diabetics (378 ± 9.0 g), at the end of the study period (P < 0.05).

**Table 1:** Blood glucose levels (mg/dl) of diabetic and non-diabetic control rats.

	Day 0	Day 1	Day 7	Day 14	Day 28
Control	108.4 ± 4.3	110.3 ± 2.5	103.8 ± 4.4	107.1 ± 7.6	86.0 ± 2.9
Diabetic	109.5 ± 3.8	518.3 ± 11.8*	555.3 ± 22.8*	549.9 ± 14.8*	576.8 ± 11.9*

Values are means ± SEM. Rats were injected with STZ (65 mg/kg) on day 0.

n = 40 rats per group.

*P < 0.05, diabetics compared to age-matched non-diabetic controls.

**Table 2:** Body Weight (g) of Diabetic and Non-diabetic Control Rats.

	Day 0	Day 1	Day 7	Day 14	Day 28
Control	203.3 ± 4.7	204.6 ± 5.8	266.6 ± 6.6	321.1 ± 8.6	378.0 ± 9.0
Diabetic	214.5 ± 3.9	200.2 ± 3.8	209.2 ± 5.6*	220.3 ± 7.3*	224.8 ± 11.0*

Values are means ± SEM. Rats were injected with streptozotocin on day 0.

n = 40 rats per group.

*P < 0.05, diabetics compared to age-matched non-diabetic controls.

**Figure 1:**
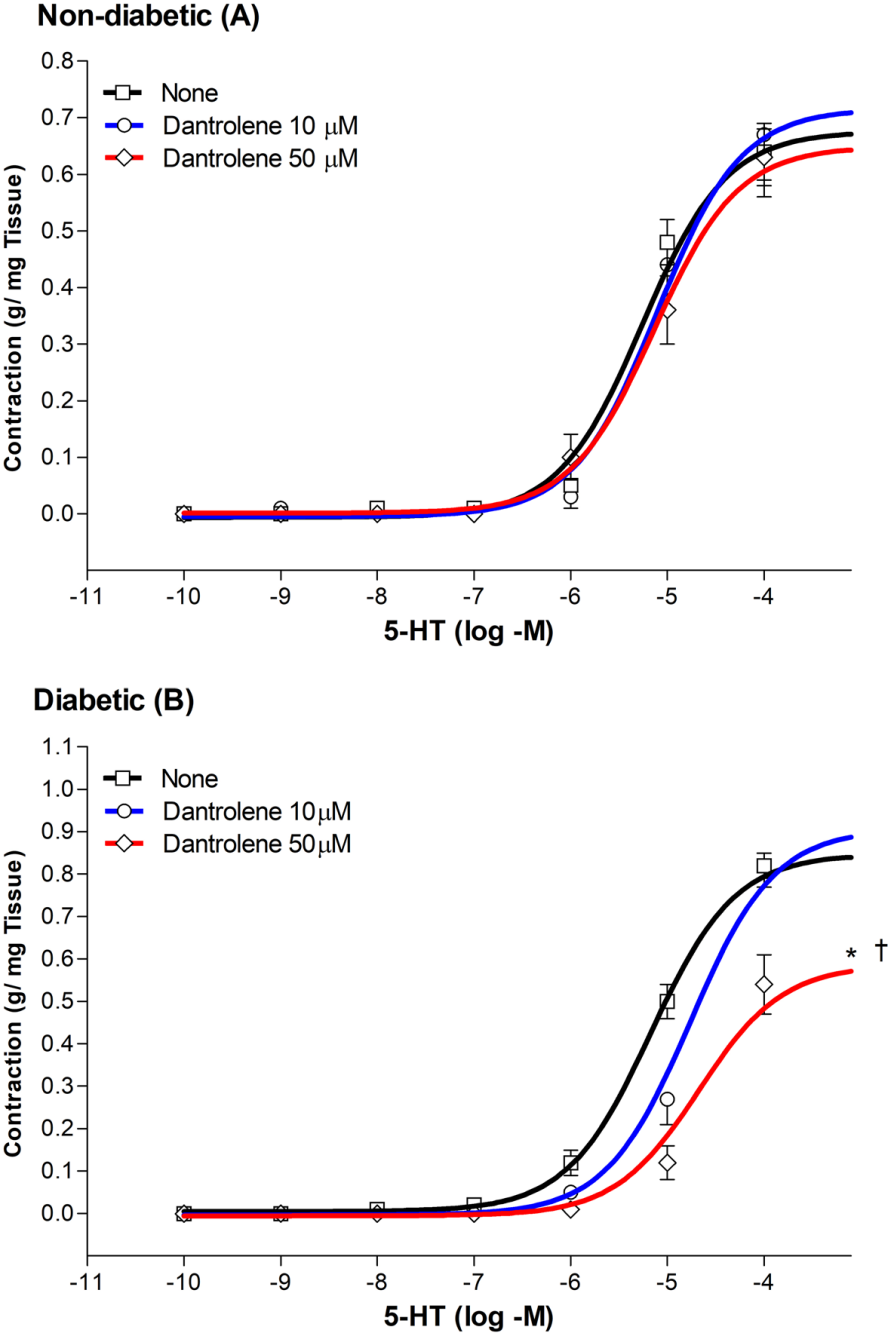
Cumulative concentration–response curves (from 0.1 nM to 100 µM) for the 5-HT-induced contraction of aortic rings from non-diabetic, (Panel **A**), and diabetic (Panel **B**) rats. Rings were incubated for a 30-min period with 10 µM and 50 µM dantrolene. The values shown are the means ± SEM of 5 to 9 animals per group. *P < 0.05, when comparing with untreated aortic rings within the same group. †P < 0.05, when comparing dantrolene 50 µM with dantrolene 10 µM-treated rings within the same group.

Figure 1 shows the effects of 10 μM and 50 µM dantrolene on the 5-HT-induced maximal contraction (Emax) in non-diabetic (Fig. 1A) and diabetic rats (Fig. 1B). In non-diabetic rats, a 30-min incubation period with either 10 μM or 50 μM dantrolene did not affect EC_50_ and Emax values (n = 7, P > 0.05). In diabetic rats, however, 50 μM dantrolene reduced Emax by 34% and increased the EC_50_ from 10.30 ± 1.18 μM to 28.15 ± 2.34 μM (n = 7, P < 0.05). Moreover, the low dose of 10 μM dantrolene also significantly increased EC_50_ to 20.39 ± 2.62 Μ (n = 7, P < 0.05).

**Figure 2:**
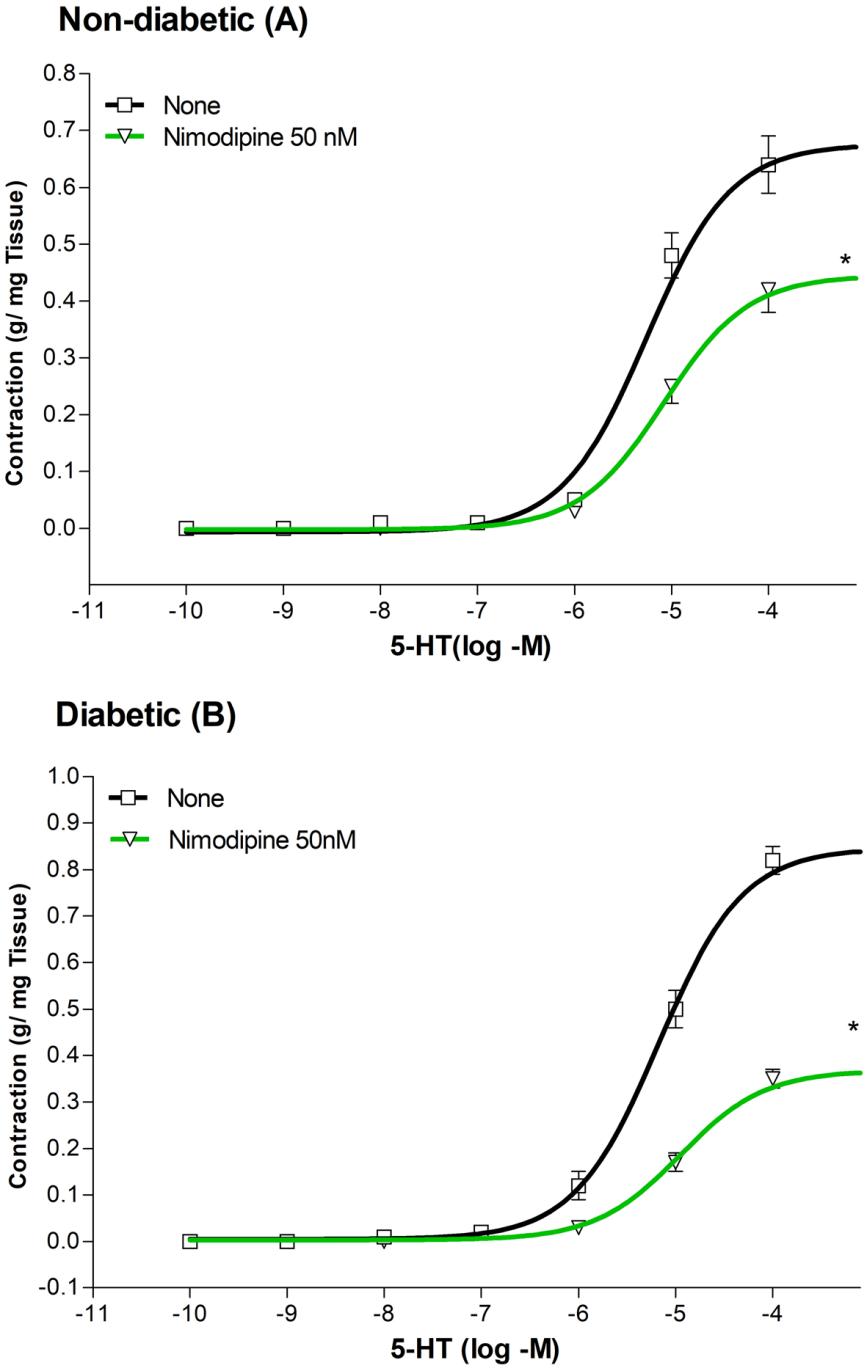
Cumulative concentration–response curves (from 0.1 nM to 100 µM) for the 5-HT-induced contraction of aortic rings from non-diabetic (Panel **A**), and diabetic (Panel **B**) rats. Rings were incubated for a 30-min period with 50 nM nimodipine. The values shown are the means ± SEM of 5 to 9 animals per group. *P < 0.05, when comparing with untreated aortic rings within the same group.

Treatment of aortic rings with 50 nM nimodipine alone, reduced Emax for the 5-HT-induced contraction by 34% in non-diabetic rats (n = 5 to 9, P < 0.05; Fig. 2A) and by 57% in diabetic rats (n = 5 to 9, P < 0.05; Fig. 2B) without modifying EC_50_ values. Interestingly, the 57% Emax reduction in diabetic rats was significantly greater than to the reduction of 34% observed in non-diabetic rats (P < 0.05; Table 3).

**Table 3:** Effects of a 30-min Incubation Period with 10 and 50 µM Dantrolene (D), 50 nM Nimodipine (N), and Both Drugs in Combination on EC_50_ and E_max_ Values for 5HT-induced Contraction in Diabetic and Non-diabetic Rats.

Conditions	E_MAX_ (g/mg tissue)	EC_50_ (µM)
**Non-diabetic rats**		
None	0.64 ± 0.05	5.93 ± 0.54
Dantrolene 10 µM	0.67 ± 0.11	8.43 ± 2.02
Dantrolene 50 µM	0.63 ± 0.05	11.61 ± 2.71
Dantrolene 10 µM + Nimodipine	0.27 ± 0.03 * ^#^	6.89 ± 0.93
Dantrolene 50 µM + Nimodipine	0.18 ± 0.02 * ^#^	15.80 ± 3.24 *†
Nimodipine	0.42 ± 0.04 *	7.49 ± 0.76
**Diabetic rats**		
None	0.82 ± 0.03	10.30 ± 1.18
Dantrolene 10 µM	0.82 ± 0.05	20.39 ± 2.62*
Dantrolene 50 µM	0.54 ± 0.07 *†	28.15 ± 2.34*
Dantrolene 10 µM + Nimodipine	0.30 ± 0.03*	9.29 ± 2.10
Dantrolene 50 µM + Nimodipine	0.22 ± 0.02 * ^#^	19.3 ± 0.66*
Nimodipine	0.35 ± 0.02*	12.40 ± 1.72

Values shown are the means ± SEM of an average of 5 to 9 animals per group.

*P < 0.05, when comparing with untreated aortic rings within the same group.

^†^P < 0.05, when comparing dantrolene 50 µM vs. dantrolene 10 µM-treated aortic rings within the same group. ≠ P < 0.05, when comparing with rings treated with nimodipine alone.

**Figure 3:**
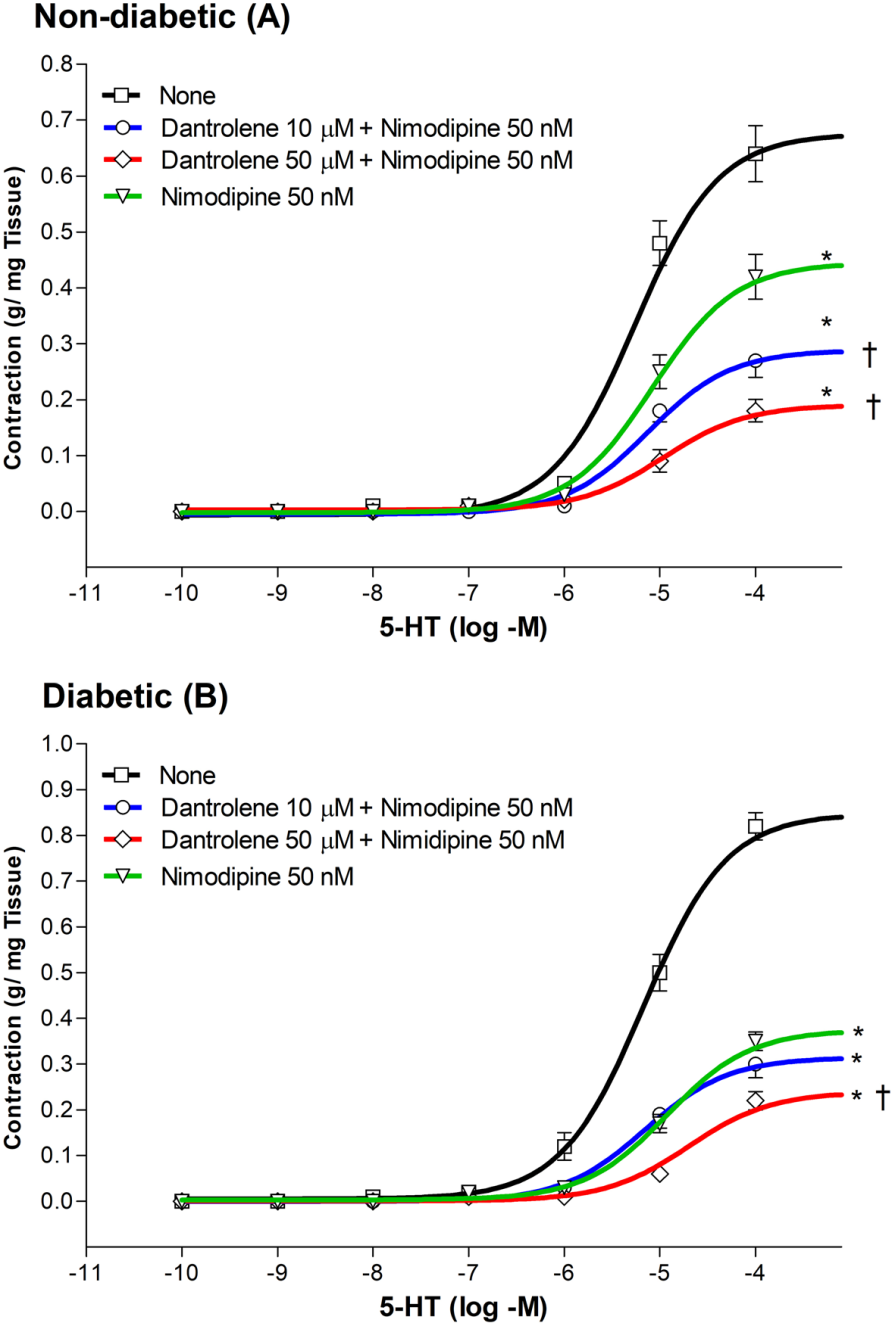
Cumulative concentration–response curves (from 0.1 nM to 100 µM) for the 5-HT-induced contraction of aortic rings from non-diabetic l (Panel **A**), and diabetic (Panel **B**) rats. Rings were incubated for a 30-min period with 10 µM dantrolene plus 50 nM nimodipine and with 50 µM dantrolene plus 50 nM nimodipine. The values shown are the means ± SEM of 6 to 9 animals per group. *P < 0.05, when comparing with untreated rings within the same group. † P < 0.05, when comparing with rings treated with nimodipine alone.

In non-diabetic rats, incubation of aortic rings with 10 μM and 50 μM dantrolene in combination with 50 nM nimodipine decreased the Emax for the 5-HT-induced contraction by 57% and 72%, respectively (n = 6 to 9; P < 0.05 when compared with untreated rings) (Fig. 3A). Similar results were found for diabetic rats, where the combined action of 10 μM and 50 μM dantrolene and 50 nM nimodipine reduced the Emax values by 62% and 73% respectively (n = 10; P < 0.05 when compared with untreated rings) (Fig. 3B). EC_50_ values, however, were only increased by combining 50 μM dantrolene with nimodipine in both non-diabetic (from 5.93 ± 0.54 μM to 15.80 ± 3.24 μM) and diabetic rats (from 10.31 ± 1.17 μM to 19.26 ± 2.82 μM; P < 0.05; Table 3).

## Discussion

We investigated the effects of dantrolene alone and in combination with nimodipine on 5-HT-induced contraction of the vasculature of type 1 diabetic and non-diabetic rats. We found that in diabetic rats, the combination of these drugs synergistically reduces the contractile response to 5-HT. The reduction of contractility is dose-dependent. Previously, we reported that these two drugs, when combined, improve endothelial-dependent relaxation and reduce PHE-induced contraction in diabetic rats^16,17^. The findings of the current study are relevant to SAH patients because bleeding is associated with higher release of 5-HT, which is an important contributor to the etiology of CVSP^26^. After hemorrhage, activated platelets release large amounts of 5-HT, which bind to the 5-HT2A receptor in VSMC and accelerate Ca2+ influx into the cytoplasm, leading to vascular contraction^27^. Furthermore, 5-HT levels are higher in diabetic than in non-diabetic patients^28^. Thus, attenuation of the effect of this vasoactive peptide may prove beneficial for diabetics who have suffered a SAH.

Our finding that, at four weeks after the induction of diabetes, the Emax value for the 5-HT-induced contraction is 28% higher in diabetic than in non-diabetic rats may partially explain why, the presence and duration of the metabolic disorder has been linked to increased risk of vasospasms after SAH in diabetic patients^29^. Similar to our finding, the contractile response to 5-HT in the superior mesenteric artery from type 2 diabetic obese (ob/ob) mice is greater than in lean controls^20^. The mechanisms underlying the augmented response to 5-HT in diabetes are still unknown. It is possible, however, that the density, affinity, or transduction pathways of the 5-HT receptors are dependent on the glycemic state. Matsumoto and colleagues^20^, found that the expression of 5-HT2A receptor in mesenteric arteries is similar between type 2 diabetic obese and lean mice, suggesting that the enhanced vasoconstriction to 5-HT in ob/ob mice is attributable to alteration in downstream pathways, rather than to upregulation of this receptor in VSM. In addition, in carotid arteries from spontaneously hypertensive rats, increased 5-HT mediated contraction is secondary to changes in multiple signaling pathways within the VSM^19^. Abnormal Ca2+ handling is also linked to the vascular complications found in diabetic patients, but the precise alterations of Ca2+ mobilizing mechanisms in diabetes are still obscure^30^. Contrary to our contention that the vascular response to 5-HT is higher under hyperglycemic conditions, Watanabe and colleagues^31^, found that diabetes leads to decreased 5HT-induced contraction in both male and female diabetic rats, when compared to non-diabetic controls. Similar contractile responses to 5-HT have been found in the mesenteric^32^ and coronary^33^ arteries of diabetic and non-diabetic rats. In addition, Ca2+ homeostasis of VSMCs and L-Type VDCC properties are altered and depend on the stage of diabetes^30^. Thus, differences in the duration of diabetes, among animal models, between the sexes, and for the particular vascular beds tested may underlie the variation reported for vascular responses to 5-HT. Indeed, the administration of 50 nM nimodipine alone was most effective in reducing Emax in the aortic rings of diabetic rats, compared to the rings of non-diabetic rats (57% vs. 34%). This finding suggests that the effectiveness of the CCB is higher under hyperglycemic conditions. Wang and collaborators^30^ found that the sensitivity of L-type VDCC to dihyropyridines is higher in VSMC from diabetic rats than that from non-diabetic rats, and that this enhanced response may be secondary to higher cAMP sensitivity due to hyperglycemia. In addition, it has been suggested that diabetes increases L-type VDCC activity in arterial VSMC^15^. Thus, the combination of increased VDCC activity and a higher sensitivity of the channel to dihyropyridines may, at least in part, explain the differences in 5-HT-induced contraction and the effect of nimodipine on VSMC between diabetic and non-diabetic animals. The implication of these findings for new therapeutic approaches to diabetic vascular complications needs further investigation.

The fact that 10 µM or 50 µM dantrolene does not decrease 5-HT-induced contraction in the aortic rings from non-diabetic rats differs from previous results reported in other vascular beds. In the basilar arteries, incubation with 10 µM and 30 µM dantrolene did not significantly affect the contractile response to 5-HT, but 100 µM dantrolene produced a significant inhibition. Moreover, in the femoral arteries, 30 µM and 100 µM, but not 10 µM, dantrolene significantly inhibited the contractile response to 5-HT^5^. Intrinsic differences in the 5-HT receptor subtypes in each tissue may partly explain these diverse findings. Specifically, the 5-HT2A receptor mediates 5-HT-induced contraction in the aorta, but 5-HT1B/D receptors mediate in the basilar artery, and by both 5-HT1B/D and 5-HT2A receptors mediate in the femoral artery^5^. In addition, differences in dantrolene doses, and in experimental methodology may also account for the different responses observed in each vascular bed.

The current primary treatments for stroke in SAH patients include surgical intra-arterial vasodilation and hemodynamic relaxation with CCBs. These treatments, however, do not effectively block the neurological deterioration observed after CVSPs^26^. The addition of dantrolene to standard therapies may improve neurological outcomes in diabetic patients because of their dual effect in reducing vascular contractility and improving endothelial- dependent relaxation throughout reduction of the lipid peroxidation markers MDA and 4-HAE in diabetic rats^16^. Thus, by acting additionally as an antioxidant agent, dantrolene may contribute to reduced neuroinflammation, which plays an important role in the pathogenesis of SAH, and improve stroke manifestations in diabetics.

Considering that type 2 diabetes accounts for the majority of the diabetic population, using the STZ-diabetic rat model of type 1 diabetes has some limitations, which include reductions in effective circulating volume and autonomic dysfunction^34^. This animal model has been used extensively, however, because it replicates both type 1 and long-term uncontrolled type 2 diabetic conditions. Nevertheless, a future study assessing the effect of dantrolene, in combination with nimodipine, on a CVSP model of type 2 diabetes would add additional insight into diabetes-related pathophysiology and the management of vasospasms.

In this study, only male rats were used, but investigating female diabetic rats would be of particular value for future study. The incidence of cardiovascular diseases increases abruptly with the onset of menopause when the protective effect of estrogen on the vascular wall is lost^35^, and by increasing oxidative stress, diabetes induces endothelial dysfunction and vascular alterations^36^. Thus, elucidating the effects of dantrolene and nimodipine on the vasculature of female diabetic rats may contribute to the design of more effective therapies for the treatment of CVSP diabetic females.

## Conclusions

To our knowledge, this study is the first to report that the combination of dantrolene and nimodipine effectively reduces 5-HT-induced contraction in diabetic rats. Salomone and colleagues^5^ found that the inhibitory effect of dantrolene on 5-HT-induced vascular contractility in the basilar and femoral arteries is substantially increased by nimodipine in normoglycemic rats. Because circulating 5-HT concentration is elevated in diabetics^28^, and the 5-HT antagonists lack of clinical efficacy in treating the cerebrovascular effects of SAH^37^, the addition of dantrolene to standard therapies with CCB may represent a promising alternative for diabetics. One limitation of this study is that the vascular effects of dantrolene and nimodipine were only tested on segments from the thoracic aorta. We expect, however, that the effect of dantrolene in inhibiting contraction will be similar for other resistance vessels because RyR1, RyR2, and RyR3 are also present in the mesenteric and cerebral arteries^38,39^, and in the cerebral microcirculation^40^. Nevertheless, Emax values for the contraction induced by 5-HT may differ because, as discussed previously, it is mediated by the 5-HT2A receptor in the aorta, by 5-HT1B/D receptor in the basilar artery, and by both 5-HT1B/D and 5-HT2A receptors in the femoral artery^5^. Therefore, it is possible that by reducing 5-HT-induced vascular over-reactivity, the combination of these two drugs improve brain perfusion after CVSPs in diabetics. Future experiments, however, are needed to assess the effects of dantrolene alone, or in combination with nimodipine, on the cerebral circulation.
